# Using a Feminist Approach to Classify Women and Girls’ Charitable Organisations

**DOI:** 10.1007/s11266-024-00687-y

**Published:** 2024-10-11

**Authors:** Lorna Dowrick

**Affiliations:** Sheffield Hallam University, Sheffield, UK

**Keywords:** Women and girls organisations, Charities, Voluntary and community sector, Non-profit organisations, Feminist, Classification

## Abstract

Women and girls’ organisations (WGOs) are a fundamental part of the voluntary and community sector (VCS) and wider society. They have a unique history and development as a sub-set of the wider VCS and have been pivotal in meeting important needs for women and girls, yet existing analysis of the VCS using data from the Charity Commission for England and Wales (CCEW) has not previously focused on WGOs. The purpose of this paper is to present a feminist approach to identifying and classifying WGOs utilising data from the CCEW register. The paper provides a detailed exploration of the methodological approach and argues that such an approach to compiling and analysing datasets is both necessary and advantageous in exposing areas of power and contestation. The paper provides an original overview of the scope of WGOs in England and Wales between 2008 and 2018 and also demonstrates benefits that can be applied in other classification work within the field.

## Introduction

Women and girls’ organisations (WGOs) are a fundamental part of the voluntary and community sector (VCS) and wider society. They have a unique history and development as a sub-set of the wider VCS and have been pivotal in developing and changing discourse around the role of women and girls within society and providing opportunities, services and activities that meet important needs. As Weldon and Htun (2013) note, the effects of autonomous organising by women have been and remain critical for, the pursuit of gender equality. It is therefore essential that there is recognition of and space for autonomous WGOs to thrive and carry out their work.

WGOs are often not included as a specific group in research about the broader VCS. One of the barriers to the inclusion of WGOs within VCS research has been a lack of reliable data about the full scope of WGOs. The CCEW compiles a register of all charities with an income over £5000 who are required by law to register. As part of the registration, organisations are categorised by type of charity; however, the list of categories that can be assigned does not include indicators for WGOs or a number of other sub-sectors (Damm & Kane, 2022). Furthermore, in one of the key sources of statistics on the UK sector, the UK Civil Society Almanac (NCVO, 2021), WGOs are also unidentified as this is based on the International Classification of Non-profit Organisations (ICNPO) developed by Salamon et al. (1996) and this system does not have a category for WGOs. Although the reasons for the lack of a specific category are not explored here, it remains clear that this systematic exclusion of WGOs as a ‘type’ of organisation is problematic and creates significant barriers to identifying WGOs and barriers for research that relies on this data. This is particularly important where data and quantitative analysis are privileged sources of information for decision-making.

This paper focuses on the methodology and challenges of the classification of WGOs as part of a doctoral research programme. It outlines how a feminist approach was used to identify and classify WGOs using regulatory data from the CCEW and argues that a specifically feminist approach to classification in compiling and analysing datasets is both necessary and advantageous for marginalised groups. First, there is an introduction to prior classification work in the VCS. Second, an overview of the feminist thought that has informed the methodology of the study. Third, the findings from applying this approach are presented including the number and types of WGOs identified. Fourth, there is a discussion of the method and results in relation to the key feminist themes and finally an outline of the applicability of this approach to identification and classification to other areas of VCS research.

An early first step in classifying WGOs is the need to determine a definition of a WGO. Defining a WGO has not been the subject of any identified extant research. Literature which includes WGOs has tended to focus on particular ‘types’ of women’s organisation based on the services they provide such as those that support people affected by domestic abuse or sexual violence (Adisa et al., 2020; Towers & Walby, 2012) or specific organisations such as Women’s Institutes (Andrews, 2015). Literature from WGOs themselves also rarely specifies a definition and can focus on describing the work that they do (National Alliance of Women’s Organisations, 2023; Rosa, 2023). One definition that can be found is reference to WGOs as organisations that are ‘led by and for women’ (Women’s Resource Centre, 2023), although there is an absence of exploration of the details and boundaries of this definition. It is useful therefore to also reflect on how broader classification within the VCS has been previously undertaken.

Significant classification work has taken place to identify the boundaries of the VCS itself. Anheier and Salamon developed an identification based on five crucial structural or operational features of non-profit organisations. Such entities had to be 1) organisations, 2) private, 3) non-profit distributing, 4) self-governing and 5) voluntary (Anheier & Salamon, 2015, p. 216). They classified units according to the types of goods or services they provide in an economic understanding of the sector based on 12 major activity groups (and a further 24 sub-groups).

It has been argued that this structural–operational definition doesn’t fully capture what is distinctive about the VCS, in particular the role of public benefit (Nickel & Eikenberry, 2016; Toepler, 2003). The definition has also been noted to exclude organisations that are not formalised and yet may include a significant proportion of VCS organisations (Billis, 1993, Toepler, 2003, and Soteri-Proctor et al., 2013). Critiques that focus on these limits of an economic understanding of the VCS and that highlight the exclusion of less formal organisations would also apply to WGOs. WGOs provide services and activities across a range of the identified groups and sub-groups such as health, advocacy and cultural activities. These services can be provided by different WGOs but also as multiple activities within one organisation. WGOs can also have a social purpose element to their work, may be informal and undertake campaign work. These features may also be applicable to many other disadvantaged groups.

Several theorists have proposed a less structured definition of the VCS that can accommodate its wide-ranging nature. Billis and Glennerster (1998) argued that third sector organisation can have comparative advantage over organisations from other sectors because of their hybridity (Billis & Glennerster, 1998; Brandsen et al., 2005). Evers also considers this fluidity as important and developed the concept of a ‘tension field’ (Evers, 1995, p. 161). All these models focus on the ambiguous nature of the VCS in recognition that organisations may exist as more or less close to the state or the market and may be more or less formal in their structure. In so doing, they can better accommodate the range of types of VCS organisation and their relative positions to the other sectors. This notion of fluidity is also extended in field theory approaches to the VCS. Macmillan proposes that instead of a single ‘sector’ the VCS is viewed as a field of organisations that are subject to continual flux and “fuzzy and permeable boundaries” (2013, p. 50). Viewing the sector as a site of multiplicity and shifting boundaries can accommodate complexity and fluidity facilitating greater recognition of change and power dynamics within the sector. Defining a sector is necessarily imbued with issues of power relations between organisations, across organisation types and in relationships with other sectors. As such, we need to consider who is involved in defining, how decisions are made and what purpose is served by the definition (Appe, 2012; Nickel & Eikenberry, 2016; and LePere-Schloop et al., 2022). Feminist approaches to the use of quantitative data highlight and centre these issues of power and contestation (D’ignazio & Klein, 2020) but in addition also emphasise unequal effects, the intersection of disadvantages and the importance of reflexivity.

### A Feminist Approach to Classification

Classification work is more closely associated with positivist traditions, raising the question of whether it is compatible with a feminist perspective. In place of a wholescale rejection of methods seen as too ‘positivist’, it is important to explore and deconstruct them to reveal their inherent subjectivity, emphasising that *all knowledge* is partial and situated (Haraway, 1988). It is argued here that identification and classification methods are devices that should be deployed in a reflexive and critical manner and the use of feminist approaches to think through and reflect on this process offers key strengths to conducting this work in more open and transparent ways. Moreover, a feminist approach can recognise, expose, and question this process and inform the way in which the research is conducted, presented and subsequently understood.

It is widely acknowledged that there are many feminisms (Humm, 1992), but this paper draws specifically on four central tenets of post-structural feminist thought including consideration of 1) gender and inequality, 2) difference and intersectionality, 3) power and language and 4) reflexivity. These are outlined in further detail below.

Central to a feminist approach is a focus on gender inequality analysing:

How gender relations are constituted and experienced and how we think, or equally important do not think about them. (Flax, 1987, p. 622).

The approach examines knowledge to uncover in what instances women and other marginalised groups are excluded, how they are represented and how their views and experiences are considered and utilised. Importantly, the views and experiences are emphasised as key starting points for enquiry through approaches which value the lived experience and knowledge of women and girls (Harding, 1991, Hill-Collins, 1990).

Difference is also a central concept to a feminist post-structural methodology. A methodology focused on interrogation or deconstruction to identify meaning and multiplicity including over time and in different contexts. Classification is influenced by the current historical, social and economic and political context in which it is undertaken (Morris, 2000), but there is also a need to appreciate variance in the detail of what is actually studied. Feminist scholarship offers a critique of binary distinctions such as mind/body, science/nature, reason/emotion and its allocation of those less valued distinctions to women (Harding, 1986; Hooks, 2014). A rejection of binaries means that a more complex approach is required that can accommodate pluralism and acknowledge the ways in which organisations may be closer or further away from different positions such as the degree to which they are formally organised or the extent to which they are led by or for women. Particularly important is the concept of intersectionality developed by Black feminist scholars (Crenshaw, 1989), where inequalities within as well as between categories are underlined, alongside an emphasis on the layering of these inequalities that create compounded effects.

For post-structural feminist approaches, language is both an important marker of power relations but also constitutive, bringing into existence that which it describes (Foucault, 2020). The language used by WGOs to describe their work within CCEW data as well as the language we use in identifying and classifying WGOs is given heightened attention by feminist approaches in recognition of its power in shaping what is presented as a landscape of WGOs.

Classification can have a performative role in influencing what is understood as a WGO sector despite only creating a partial view, where some things are made visible while others are not. This power to create visibility is also therefore a principal part of this process of identifying and classifying WGOs. This power can be both positive and negative and one that can bring increased recognition but also surveillance (Nickel & Eikenberry, 2016), create unity as well as generate division and exclusion. It is therefore essential to take a reflexive approach to who is classifying, for what purpose and what is the impact of the process.

It is argued that this application of feminist theory can be applied more broadly to the field of classification of VCS organisations. It can offer additional important ways to reflect on the process, draw attention to unequal effects, enhance transparency and highlight difference alongside similarity. In particular, the themes and key questions can be applied to other classification projects prompting reflection on how they can be adapted to a specific approach for that study. The themes, key questions and how they have been operationalised in this classification process are summarised in Table 1.

**Table 1:** Feminist approach to classification of WGOs

Feminist theme	Key questions	Approach
Gender and inequality	What is the role of ‘gender’ in this topic? How are marginalised groups represented or excluded in the classification process? How are the views and experiences of marginalised groups considered and utilised?	Noting and exploring the absence of existing classification mechanisms for WGOs Reflection and articulation of how women and WGOs are defined Critical analysis of the process of classification Exploration of the WGO proposed definition of ‘by and for’ women and girls Use of WGO descriptions within CCEW fields to create categories and sub-types of WGOs
Difference and intersectionality	How is difference accounted for? What are the effects on groups facing multiple disadvantages? How can intersectionality be represented in the classification?	A rejection of binaries in favour of fluidity and range A broad approach to including organisations Exploration of differences within the data Creation of categories and sub-categories that include exploration of multiple facets of an organisation
Power and language	What role does language have in the classification? What are the effects of the terms of classification?	Acknowledgment of contestation of categories Reflection on what language is used and in what ways to describe categories Recognition of constitutive effect of categories Exploration of classification as a source of negative and positive effects
Reflexivity	Who is undertaking the classification? For whom and for what purpose is the classification taking place?	Reflection on the role of the researcher in creating the classification and the impact of the process Transparency of decision-making Exploration of the purpose and who benefits from the classification

## Method

To define a WGO, it is first necessary to acknowledge that the category of ‘woman’ itself is open to contestation. Gayatri Spivak has argued that feminists need to rely on ‘strategic essentialism’ where the term ‘women’ is used to bring unity around a common goal while acknowledging the temporary and contingent nature of the term (Spivak, 1988). The terms woman and women were used in this study to denote a category, but it was also acknowledged that this category can encompass a variety of positions and as such organisations which specifically support people with the topic of gender identity were included. Following the ‘strategic’ use of woman above, this same use was also applied to defining WGOs. As stated above, it has been proposed that WGOs are those organisations which are ‘by and for women and girls’ (Women’s Resource Centre, 2007). A focus on ‘for women and by women’, however, can also lead to a narrow understanding of WGOs. If adhered to strictly, it would potentially exclude organisations that many would see as being key to a ‘women’s organisation sector’. The delineations between organisations that are either by or for women, both, or neither, are in practice less clear than at first sight. This study therefore moved away from trying to establish *the definition* of a WGO and instead allowed for a wider ‘field or landscape of organisations’ that replaces ‘led by and for women and girls’ with a broader ‘predominantly led by *and/or* for women and girls’. A landscape takes account of the varying degrees to which organisations are run entirely by women as well as different degrees to which they are ‘for women’. It also gives greater visibility to the range of organisations who predominantly benefit women. This provided a starting point for an iterative approach that built on and worked with the descriptions and information within the register to reflect on whether an organisation may be a WGO.

The register of charities provides the most comprehensive set of data available for voluntary sector organisations in England and Wales. There are, however, several important limitations, and a feminist approach reminds us to reflect on what the selection of a dataset will mean for the inclusion and exclusion of certain groups, particularly those already facing additional disadvantage. By focusing on organisations that are registered, we are unable to capture *all* potential WGOs. Smith described this as a ‘flat earth’ approach to mapping the non-profit sector (Smith, 1997) where mapping is carried out in a way that captures what is available and not what is known to exist. The use of charity register data excludes several organisational forms that may particularly be part of a women’s sector such as unregistered informal and smaller organisations (Vacchelli & Kathrecha, 2013), campaign alliances that are not registered charities, organisations using other legal forms such as Community Interest Companies (CICs) and a bias towards larger groups and more well-resourced organisations. There will also be projects for women located within and existing with varying degrees of autonomy from organisations that serve multiple needs of a community. As such, it is important to recognise that the process of identification and classification using the CCEW register will always lead to an incomplete account of WGOs and other VCS organisations.

Despite these limitations, the CCEW register arguably provided the strongest starting point for identifying WGOs and from which further work can be developed. The CCEW register has detailed information about charities and provides data each year. It has the potential to provide longitudinal information about changes to the sector across longer time periods. Furthermore, as there are no datasets available that specifically identify WGOs within them, identification using keyword searches in textual data is both a useful alternative (Damm & Kane, 2022; Lepere-schloop et al., 2022; Litofcenko et al., 2020) and makes it possible to acknowledge and gives scope to explore the role of WGOs in identifying themselves through the language they use in the data. The name, charitable objects and activities fields within the CCEW register all provided information about organisations which were used to ascertain whether they were likely to be a WGO. Prior research had created a sample of CCEW data for Yorkshire and the Humber for 2018 which was used to develop a set of keywords used by WGOs (Dowrick, 2018). All organisations located in Yorkshire and the Humber in CCEW data for 2018 were selected for review (*n* = 12,007), and the name, activity and purpose fields were all examined individually to create a broad dataset of possible WGOs (*n* = 384). From this dataset, a list of key words was compiled with the aim of creating a keyword list which if applied using automatic keyword searching would identify all of the organisations found through the manual process. The final keyword list was extensive and contained over 50 words. The keyword search was applied to the dataset of registered charities for 2008 to 2018 (CCEW, 2019) in an automated process using the statistical software package SPSS.

Relying purely on this list of words inevitably generated a large rate of false positive results, due to the frequent use of these keywords by organisations in general and who are not predominantly for women; for example, an organisation may include the word ‘women’ in its description without being a women and girls organisation. The aim of the search was to ‘cast the net wide’ and ensure that as broad a range of organisations as possible could be explored to limit the exclusion of groups who may be a WGO. A further layer of analysis was essential to make judgements about whether an organisation was ‘predominantly for women’. This layer was based on close reading of the descriptions in the text and where necessary further desk research to seek additional information from charity websites.

Research by Litofcenko et al. (2020) used a keyword search focused on the names of charities and found that this was able to provide sufficient identification of charities. It is argued here, however, that while more time-consuming, it is important to use a broader approach for a number of reasons. As Litofcenko et al. (2020) and Jung et al. (2018) note, charity names do not always reflect their purpose and may contain few clues to the nature of the organisation. Close examination of a broader range of data provided better clues to identify organisations who may not necessarily use clear terms such as ‘women’ or ‘girls’ in their text. For example, alternative terms such as sister, widow, ladies and more are all found in descriptions of WGOs while organisations such as ‘Inner wheel’ organisations run by women often did not have a reference to women and girls in their title. Particularly for WGOs, there are also many alternate ways to describe women and girls which makes the identification of WGOs difficult.

An iterative approach was used to move back and forth between the proposed definition of a WGO and the organisations that were being categorised. This led to adjustments and refinement of the boundaries of the category. It was possible to identify a range of organisations that may not explicitly be ‘for women’ but which on closer examination it was found that their purposes and aims were predominantly for women, and they were therefore included. Some organisations, for example, described themselves as supporting ‘people’ affected by domestic abuse or sexual violence rather than women and girls, illustrated by one charity from the register, which states:

The Charity is established to relieve distress and suffering amongst people living with or fleeing from, or at risk of, Domestic Abuse (emphasis added). (CCEW, 2019).

These organisations were included as it was assumed that the majority of beneficiaries would be women and girls as they form the majority of people affected by issues such as domestic abuse and sexual violence (Office for National Statistics 2022a, 2022b). Organisations for lone parents, for example, are also organisations who are often mostly supporting women, since women constitute the majority (around 86 per cent) of lone parents (Dromey et al., 2020). However, organisations that operate in an area which predominantly affects women such as ‘domestic abuse’, but the details stated that it was an exclusively for men service—were excluded. Descriptions therefore warrant closer inspection, and a focus on difference and detail is important.

A wide view of WGOs also highlights organisations that may not have previously been included such as those providing funding to individual women. It therefore brings the possibility for greater acknowledgement of those organisations and the potential to explore new areas for collaboration between WGOs about addressing the needs of women and girls.

Ultimately, a boundary must be found to create a final dataset, but a feminist approach highlights that it is both possible and important to stretch and explore the edges of a boundary because there are many overlaps and ‘fuzzy’ edges as organisations do not neatly fit within one space or another (Brandsen et al., 2005; Macmillan, 2013). This applies not only to WGOs but is also an important consideration for other types of organisation.

## Results

This section will now outline the key findings generated from the feminist approach. First examples of text from the CCEW data will be discussed to highlight key findings from the identification process, followed by a presentation of the number and types of WGOs identified.

Charities typically described their activities in ways which underlined their purpose. Being ‘for women’ was a key feature, often including a list of a broad range of activities that they undertake to achieve this aim. For example:

(charity) is run by women for women to provide appropriate training, information and support, which addresses the needs of women within different communities… (CCEW, 2019)

Provision of support, advice and accommodation to women and children living with, escaping or recovering from the effects of domestic or sexual violence. (CCEW, 2019)

Although WGOs may be grouped together into a sub-category, they also occupied a range of positions in relation to their values and approaches to providing the services and activities. In the first example below, the project is focused on the aim of actively encouraging women to leave sex work:

the … project aims to offer friendship, advice and opportunity for change to women working in prostitution…with an ultimate goal of seeing them leave this lifestyle… (CCEW, 2019)

In the second example below, the charity highlights the promotion of safety while recognising choice for females engaged in sex work.

(charity) works with female sex workers to promote sexual health, wellbeing and personal safety whilst offering choice, support and empowerment… (CCEW, 2019)

WGOs may also target support to specific groups of women rather than provide for all women and girls such as those for women affected by a particular issue or living in a specific area. Organisations for Black and Minoritised Women and Girls (OBMWG), for example, have important differences between them in purpose and activities. In terms of the women they support, some organisations may be for women and girls from specific backgrounds or for women and girls facing a common issue. These differences are not visible when they are categorised together in one group.

Establishing if a WGO was ‘by women’ was challenging due to a lack of publicly recorded data for board membership or leadership by gender. It is likely therefore that leadership of included organisations may not be exclusively comprised of women, and it is expected that there are a range of organisations in the resultant dataset with varying numbers of women within the leadership.

There were also organisations explicitly led by women but for a range of other beneficiaries such as women’s fundraising groups. These organisations were identified through their charitable aims and objects as being ‘women-led’ organisations. There may also have been organisations that are women-led but did not include reference to this within the relevant fields, and as such, it was not possible to identify them, and they will therefore have been excluded.

Once the keyword and identification process had been completed, a total of 12,473 WGOs were found to have existed within that period (Dowrick, 2023). Table 2 outlines the types of organisations identified, along with an additional layer of sub-types of organisations. Sub-types were added to allow for a more nuanced insight, discussed further below. To aid analysis as there were very small numbers of organisations in these sub-types, there were also several sub-types of organisations grouped into the ‘women and girls facing additional disadvantages’ category. The table importantly indicates the main exclusions for each category.

**Table 2:** Types and number of WGO between 2008 and 2018

Organisation types and total number	Description	Sub-type	Exclusions
Ending violence against women and girls (EVAWG) (828)	Organisations seeking to end VAWG, and/or support women affected by VAWG	Women only EVAWG EVAWG Plus (organisations that also support people who are not women) Refuges Organisations that support women affected by trafficking	Housing organisations that are not refuges Anti-trafficking organisations that are not women specific International EVAWG organisations
OBMWG (444)	All OBMWG	Health General welfare Social EVAWG Education and employment Gender equality Disability Other	Organisations which are not predominantly for Black and minoritised women and girls
Health (591)	Organisations supporting women with health issues (prevention, fundraising, or treatment/support)	Breast or ovarian cancer organisations Family planning organisations Women’s health centres or health projects	Organisations supporting all people affected by cancer Organisations working outside the UK only
Social (8207)	Organisations that mainly provide social activities for women	Women’s institutes Girlguiding Townswomen’s guild Inner wheel Other social groups	Organisations providing a range of services not just primarily social activities
Education and employment (456)	Organisations providing education opportunities for women and girls or supporting women and girls with employment or training	None	Schools
Sports and arts (117)	Organisations that provide sporting or arts opportunities for women and girls	Sport Art	Primarily social organisations which include some arts or sports within their activities
Funding and resources (621)	Organisations that provide funding to WGOs or funding to individual women WGOs that only own buildings	Funding organisation Buildings	Organisations that have a building, but it is not their sole/primary purpose
Gender equality (69)	Organisations that raise awareness about and campaign for gender equality	None	Organisations that campaign for gender equality, but it is not their sole or primary purpose
Housing (261)	Organisations that provide housing for women	None	Refuges and emergency accommodation relating to EVAWG
Giving to others (44)	Organisations that are run by women but benefit a range of beneficiaries	None	Inner wheel organisations
Women and girls facing additional disadvantages (128)	Organisations supporting women and girls facing additional disadvantages	WGOs that focus on: Gender identity and sexuality Women and girls with a disability Women and girls affected by the criminal justice system Women who are sex workers Lone parents	Organisations that offer wider support to all LGBTQIA + individuals Provision for anyone affected by the criminal justice system Organisations supporting only male sex workers
Faith or moral welfare (220)	Organisations which are based on the promotion of or education about a religion or describe themselves as ‘moral welfare’ organisations for women	None	Faith organisations that only operate internationally
General welfare (155)	Organisations providing a range of support to women and girls. This can typically include health and well-being, advice and support and social activities	None	Organisations providing primarily one main activity
International (332)	Organisations working internationally	None	Organisations operating primarily in the UK

## Discussion

The discussion is organised using the key themes outlined in this feminist approach 1) gender and inequality, 2) difference and intersectionality, 3) power and language, and 4) reflexivity.

### Gender and Inequality

A focus on gender and other inequalities foregrounds the lack of adequate recognition and inclusion of WGOs and other marginalised groups within the current classification systems such as the ICNPO and the CCEW register classification codes. WGOs and many other organisations for marginalised groups and communities are not visible within these current systems although work is now underway by the CCEW to review their classification categories (CCEW, 2022). The lack of visibility for WGOs effects their ability to be recognised as a group and fails to acknowledge the importance of the range of services or beneficiaries as defining features of the organisations. This reinforces inequality in a number of important ways. An absence of systematic knowledge reduces the ability to be able to use the data to research and analyse important changes over time, seek recognition and secure resources to carry out their work but also reduces visibility for WGOs in amplifying the voice of their beneficiaries.

WGOs registered as charities meet the five definitional criteria outlined by Anheier and Salamon (2015), but this analysis confirms the findings of prior research (Nickel & Eikenberry, 2016) that as the definition is based on an economic understanding of the sector the sub-categories used in the definition do not fully capture the wide range of social benefits and opportunities that they provide. The social impact of WGOs is an important feature of these organisations, and the results show that social organisations constitute not only the majority of WGOs but that WGOs often have social activities as both a key element of and means to achieve their work. They are often providing multiple services for women and girls as demonstrated by the ‘general welfare’ classification. Furthermore, the close examination of the language used by WGOs to inform the definitions shows that being an organisation *for women and girls* can be an important marker for the organisations. An identity as being for women and girls (the who) is a defining feature rather than the services and activities (the what). Their identity is not adequately recognised within current systems.

### Difference and Intersectionality

Categories were developed based on an iterative process using themes that emerged from the descriptions that charities gave of their work. Each WGO was assigned to one main category, based on their description such as an organisation providing social activities or supporting women and girls with health and well-being. However, the categories can only be seen as indicative. There were many versions of the categories as a balance was sought between sufficient detail to make categories coherent and not so many categories to make comparisons between them too complex and unwieldy. The selection of one sub-type for an organisation was problematic as the nature of many WGOs means that they may work in more than one category area such as an organisation supporting women with both health and education and training. To reflect this, a category for ‘general welfare’ organisations was created which encompassed a variety of organisations for women offering multiple services including but not limited to women’s centres.

Change is continual for both WGOs as a group and as individual organisations. WGOs may move over time towards or away from being ‘by women and for women’; for example, organisations that may have previously offered services to just women may now offer services to men. This can be in response to the external environment or pressures such as wider cultural, social, economic or political shifts but may also be a consequence of internal organisational dynamics such as a leadership change. A continual cycle of new organisations joining the sector and others ceasing to exist adds to the fluidity within the sector. It is important to note these dynamics *within* any ‘boundaries’ of the sector.

All organisations that meet the ‘predominantly by and for’ criteria were included in the dataset produced in this study, but there is not a universal ‘woman’ to whom all organisations for women can benefit. An exploration of the CCEW data revealed significant nuance as WGOs serve different purposes and agendas and there can be discord or competing approaches between organisations of a similar sub-type. This may also change over time. WGOs may be more or less open to all women, and others may have more or less of a commitment to gender equality issues. Heterogeneity within sub-types also requires acknowledgement. Organisations assigned to a sub-type with different goals, values, or ways of working were placed within the same sub-category suggesting a degree of alignment, but this concealed important differences.

### Power and Language

Every effort was made to try and build the categories from the data and the descriptions by WGOs, and this process was inevitably subjective and exclusionary, but close examination of the descriptions also offered a deeper understanding of how organisations describe themselves and their work. It can signal alignment within a wider group of WGOs where there may be strong historical roots or connections, a shared identity and common purpose. It is also a means of accessing recognition and resources as a group, for example, where funding may be targeted towards ‘women’s organisations’. Use of non-gender specific terms such as ‘people’ may lead to a separation or exclusion from the group; similarly, using exclusively the language of ‘women and girls’ may have exclusionary effects on people who may identify with other gender identities. This choice of language may be both conscious and unconscious but has lived effects both for the organisation and those it seeks to either offer or decline to support. It is important to recognise for classification purposes as it can give clear indications of an organisation’s focus and without paying attention to the wider social, political context in which this language is used can lead to the exclusion of particular types of groups. For example, focusing on only organisations which contain the terms ‘women’ and/or ‘girls’ may exclude key organisation types who have chosen to describe their beneficiaries in alternate ways.

Classification as a specific group can bring increased visibility for WGOs and shape how the landscape is understood. The organisations included in this research already have a degree of visibility as they are registered with the CCEW in a publicly available dataset, but as stated earlier they are also less visible than many other types of organisations as they are entities who as a group are dispersed within other categories used in this regulatory dataset. Visibility, however, is about more than just being seen. It infers that there is someone to be seen by (visible to whom?) and a desire to be visible (visible for what purpose?). As Chow notes:

Less a matter of becoming physically visible than a matter of attaining discursive attention and recognition, of which being visible simply serves as a metaphor. (Chow, 2010, p. 64).

This visibility can have both positive and negative effects. Registering with the CCEW means that organisations are both subject to constraint through increased scrutiny (Foucault, 2020; Nickel & Eikenberry, 2016) but are also able to gain recognition and access to resources. Greater prominence involves risks such as increased alignment with state goals and consequently a decline in independence and the ability to work towards more radical gender equality goals. This is significant when independence has been noted to be critical for pursuing gender equality goals (Weldon & Htun, 2013). These issues of visibility therefore also feed into wider debates about the VCS’ independence from and relationship to the state (Egdell & Dutton, 2017; Milbourne & Cushman, 2015). In identifying and classifying organisations in new ways, an understanding is needed of balancing the potential benefits for organisations of becoming visible against these possible consequences.

### Reflexivity

The resultant representation can also be interpreted (both intentionally and unintentionally) in different ways and used for a variety of purposes for example to justify varying aims or decisions. It is important therefore to be reflexive about what is created and how it can be used. As Lather notes, there is a requirement to examine the implications of the researcher’s actions.

What would a sociological project look like that was not a technology of regulation and surveillance? (Lather, 1991, p. 15)

It is also significant to recognise that increased visibility has been advocated by a selection of WGOs themselves. They are frequently aware of where state and WGO agendas are not compatible and have consistently raised this as an issue (Dowrick, 2023).

This feminist approach gave space to reflect on how different subjective viewpoints and approaches may provide nuanced and important differences in what is shown and the implications of this for the ‘landscape’ that is presented. While there may be some common notion of which organisations are WGOs, there is also likely to be considerable contestation about the boundaries of the term and what is included. As Appe reflected in her research, different mappers will map different things based on their own objectives (Appe, 2019). This role of the researcher in shaping the ‘reality’ that they present is critical to note as it is important both for transparency and scrutiny while also revealing the power exercised by the researcher. A process which included explicitly highlighting exclusions and limitations enables others to understand, use or develop alternate views of the landscape through using the data to create new combinations of groups, seek additional inclusions and/or offer new critiques.

### Broader Application of the Approach

Creating, naming and analysing this dataset have constructed a landscape of a potential sector of WGOs that may or may not be widely agreed upon. Now created, this dataset could be used for further analysis and research and may have a performative effect on how the sector is defined, measured and counted with the potential for a range of consequences for WGOs. The same may be true for similarly disadvantaged groups.

The feminist approach outlined in this paper can be readily adapted for use in other classification projects through the application of the themes and key questions to new contexts. This would facilitate a focus on issues of gender, differences in power, marginalisation and exclusion and representation which also centres a more reflexive approach to the process of classification and its effects.

Datasets can be created and made available to organisations for their use and reference. The ability to be able to track changes and have information about the landscape of disadvantaged groups offers possibilities for making new connections between organisations, lobbying for resources and identifying historical developments. It offers the opportunity for disadvantaged organisations to be ‘as visible’ as other sectors and in this way allows greater recognition, where organisations can be better understood and acknowledged for the breadth of work that they do. It also brings the possibility of increased support and resourcing as the issues that they face as organisations and raise for those they support become potentially better understood. Importantly, a feminist approach alerts researchers to the complexity of creating representations.

## Conclusion

The study began with a broad range of keywords to build an iterative picture of WGOs, exploring the edges of what may or may not be within its boundaries. This raised interesting and critical questions about the process of inclusion and exclusion and its consequences. Using a post-structural feminist approach to both direct the classification process and importantly reflect on its consequences highlighted the complexity of creating representations and centred the issues of (in)equality where a necessary process of inclusion and exclusion takes place. Furthermore, the approach underlined both the subjective nature of the classification and the capacity for alternate classificatory judgements. The focus on difference directs attention to exceptions so that a more nuanced and complex understanding is achieved. Ultimately a classification process led by a feminist approach can be usefully applied to many other areas of VCS research where the diversity of the sector is well known and where it has been noted that there are many sub-groups of organisations with whom organisations may more closely identify than a broader VCS sector (Macmillan, 2013). A feminist approach ensures that researchers take account of any unequal impact that the identification and classification may have, particularly for disadvantaged groups.
